# A decreased ratio of height of lateral femoral condyle to anteroposterior diameter is a risk factor for anterior cruciate ligament rupture

**DOI:** 10.1186/s12891-020-03440-w

**Published:** 2020-06-23

**Authors:** Ruibo Li, Xingyue Yuan, Zhi Fang, Yuehong Liu, Xi Chen, Jianjun Zhang

**Affiliations:** 1 Department of Orthopaedics, People’s Hospital of Deyang City, No. 173, section 3, North Taishan Road, Deyang, 618000 Sichuan Province China; 2 Medical College, Qingdao University, Qingdao, 266000 Shandong Province China

**Keywords:** Anterior cruciate ligament, ACL, Knee, Lateral femoral condyle, Femur, Risk factor, DR

## Abstract

**Background:**

Studies have shown that the spherical shape of the lateral femoral condyle has a clear relationship with the relative axial movement of tibiofemoral joint and the anterior cruciate ligament (ACL) rupture. The purpose of this study was to describe the distal curvature of the lateral femoral condyle by ratio of height of lateral femoral condyle to anteroposterior diameter (HAPR), and evaluate its correlation with ACL rupture.

**Methods:**

A retrospective case-control study of 64 patients was conducted. Two age-and sex-matched cohorts (each *n* = 32) were analyzed: primary ACL ruptures, and a control group consisting of isolated meniscal tears. On the radiograph, the distance from the intersection of the axis of the distal femur and the anteriorly diameter of the lateral femoral condyle to the lower point of the lateral femoral condyle divided by the anteriorly diameter of the lateral femoral condyle is HAPR. The HAPR was measured by digital radiograph imaging systems (DR) to quantify femoral sphericity. Cutoff values were defined; and diagnostic performance of the risk factors was assessed. Meanwhile, we measured the posterior tibial slope (PTS) on radiograph and compared the two methods to evaluate the significance of HAPR in predicting ACL rupture.

**Results:**

A total of sixty-four patients who met the inclusion criteria were included in the final analysis (32 with primary ACL rupture, 32 controls). The HAPR was smaller in the knees with primary ACL rupture (0.31 ± 0.02) than that of the control group (0.33 ± 0.02) (*p* < 0.01). The PTS was bigger in the knees with primary ACL rupture (8.18 ± 2.77) than that of the control group (6.61 ± 2.85) (*p* = 0.036). The AUC of HAPR was bigger (0.825; 95% CI, 0.72–0.93) than that of PTS (0.675; 95%CI, 0.85–0.81). The calculated cutoff of HAPR of 0.32 (Youden index, 0.56) was associated with an increased risk for ACL rupture, with sensitivity of 75% and specificity of 81% to predict an ACL rupture.

**Conclusions:**

This study showed that a decreased HAPR is associated with an ACL rupture, and the decrease of HAPR was more significant in predicting ACL ruptures than the PTS. This helps clinicians identify susceptible individuals who may benefit from targeted ACL rupture prevention counseling and intervention.

## Background

Anterior cruciate ligament (ACL) ruptures are the most common knee ligament injuries, with an incidence of about 68.6 per 100,000 person-years [1]. Because of the heavy social and economic burden brought by ACL injury, it is very important to identify and prevent ACL injury [2]. The nature of ACL rupture is multifactorial, including tibial slope, femoral notch size, knee valgus and other anatomical factors, as well as body mass index and environment, which have been confirmed by many studies [3, 4].

Among the anatomical factors, the influence of the anatomic variance of the lateral compartment has recently attracted considerable research interest [5]. Some studies have shown that the spherical shape of the lateral femoral condyle has a clear relationship with the relative axial movement of tibiofemoral joint and the ACL rupture [6, 7]. The purpose of this study was to describe the distal curvature of the lateral femoral condyle use ratio of height of lateral femoral condyle to anteroposterior diameter (HAPR), and to evaluate its correlation with ACL rupture. It was hypothesized that a decreased ratio of height of lateral femoral condyle to anteroposterior diameter would correlate with an increased risk of ACL injuries.

## Materials and methods

### Study design

This retrospective cohort case-control study was conducted in the department of orthopedics at Deyang People’s Hospital. This retrospective study involving human participants was in accordance with the ethical standards of the institutional and national research committee and with the 1964 Helsinki Declaration and its later amendments or comparable ethical standards. The Human Investigation Committee (IRB) of Deyang People’s Hospital approved this study.

**Fig. 1:**
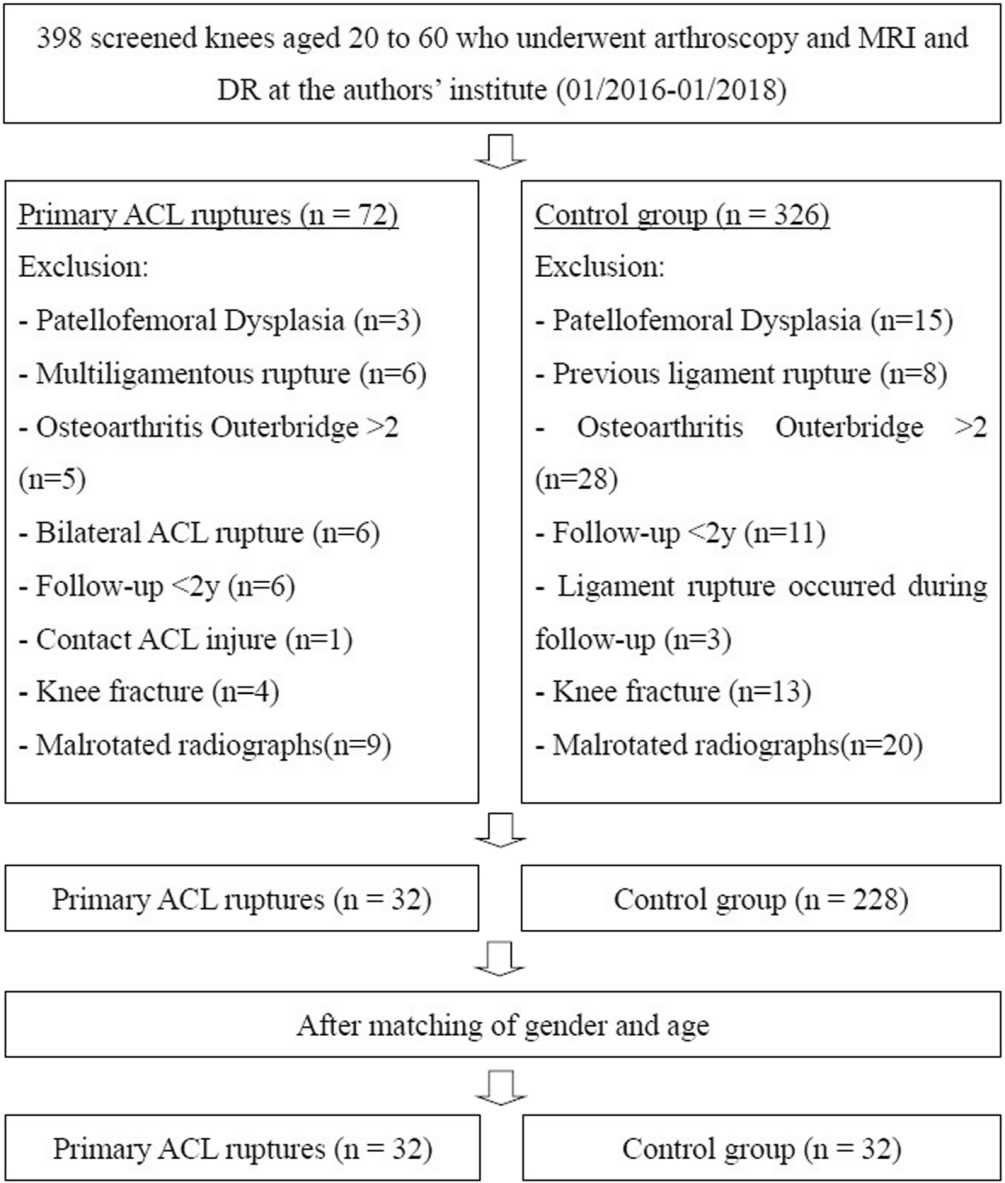
Flowchart and eligibility. ACL, anterior crucial ligament; MRI, magnetic resonance imaging; DR, digital radiograph imaging systems

A retrospective analysis was performed on 398 patients aged 20 to 60 who underwent arthroscopic knee surgery for non-contact simple ACL rupture or isolated meniscus injury in author’s hospital from 2016 to 2018, and they were divided into the following 2 groups: (1) primary ACL ruptures, (2) a control group consisting of patients with isolated meniscus injury without concomitant ligament lesions and signs of patellofemoral dysplasia. The ACL rupture cases were matched to control group according to sex and age and then assessed for the eligibility criteria listed in Fig. 1. The minimum follow-up time was 24 months for all the patients. All patients underwent preoperative digital radiograph imaging systems (DR) and magnetic resonance imaging (MRI) examination, and were read by senior radiologists and surgeons to determine the presence of anterior cruciate ligament rupture or meniscus rupture. All patients included in the study underwent arthroscopic knee surgery, and the presence of combined injuries was reconfirmed intraoperatively.

### DR measurements

The lateral knee radiograph was taken at 30 degrees flexion and the femoral condyle overlap radiograph included the area between the lower half of the femur and the upper half of the tibia, including the patella and posterior joint boundary. We referred to Thomas r. Pfeiffer ‘s method to determine the acceptable threshold for femoral condyle overlap [8]. Thirty patients were randomly selected from the population of patients after primary screening. Two independent, blind observers measured the degree of overlap (i.e., malrotation) and HAPR of the lateral femoral condyle. Intracalss correlation coefficient (ICC) analysis was performed, including varying degrees of malrotation (0-3 mm, 0-6 mm, 0-9 mm, and 0-greater than 9 mm), to determine the reliability of each group of observers. The ICC threshold of 0.7 was acceptable. The interobserver reliability ICCs were 0.86 at 0 to 3 mm, 0.74 at 0–6 mm, 0.68 at 0–9 mm, and 0.62 at 0 to greater than 9 mm. Therefore, we determined the threshold to be 6 mm (ICC = 0.74), and radiographs of the femoral condyle overlap < 6 mm were included in the study.

**Fig. 2:**
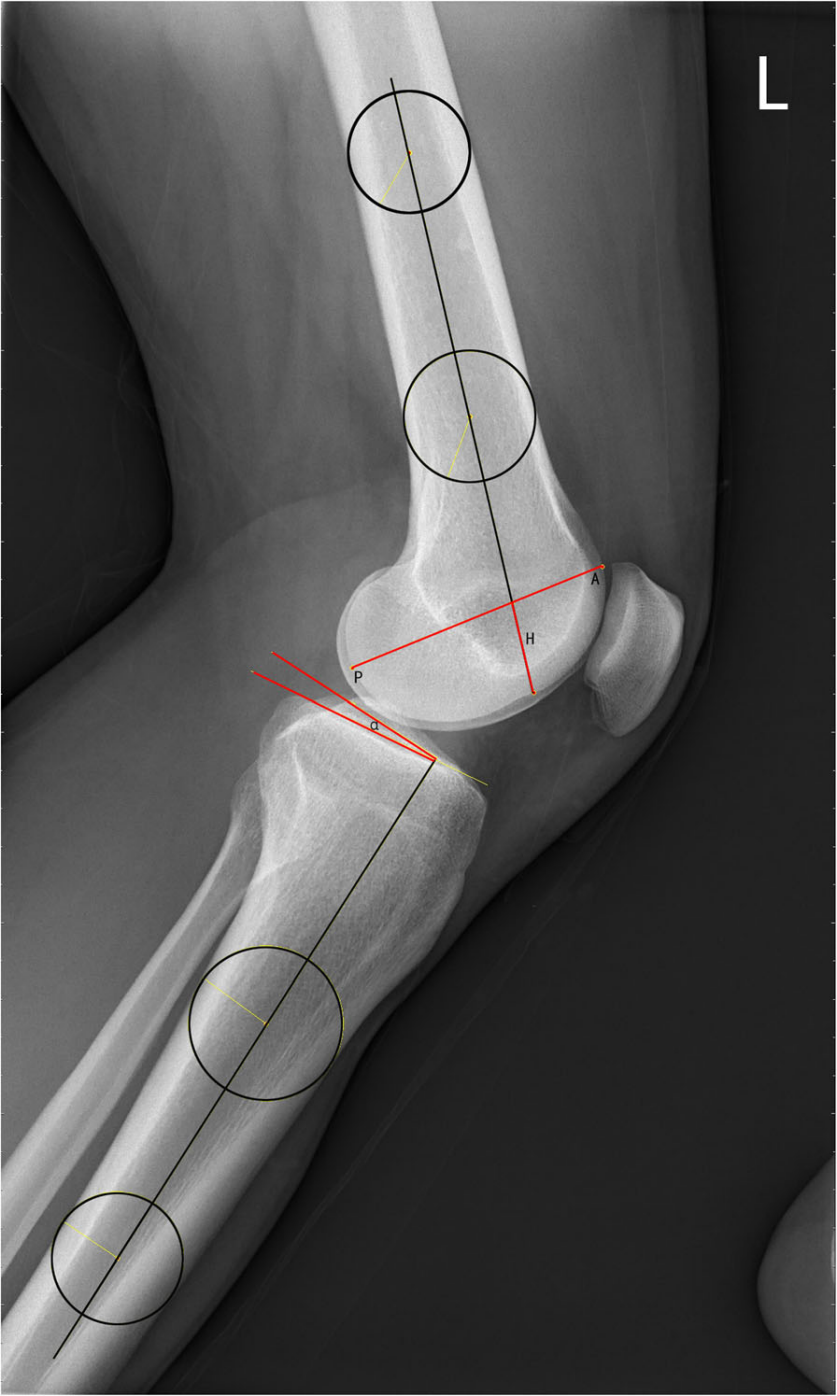
The distal circle is 5 cm from the distal end of the femoral condyle, and the proximal circle is 5 cm from the distal circle. The line passing through the center of the two circles was considered the long axis of the distal femur. The axis of the lateral femoral condyle of the femur was then determined by drawing a line between the most posterior and most anterior points of the lateral femoral condyle (AP). The distance from the intersection of these two lines to the most inferior point of the lateral femoral condyle defined as the height of the lateral femoral condyle (H). HAPR = *H/AP.* The proximal circle is 5 cm from the tibial plateau, and the distal circle is 5 cm from the proximal circle. The line passing through the center of the two circles was considered the long axis of the proximal tibia. The post tibial slope (PTS, α) was defined as the angle formed by the axis that passes through the diaphyseal centre and a line parallel to the tibial plateau

To determine the long axis of the distal part of the femur, two circles separated by 5 cm were centered on the femoral shaft [8]. The distal circle is 5 cm from the distal end of the femoral condyle, and the proximal circle is 5 cm from the distal circle. The line passing through the center of the two circles was considered the long axis of the distal femur. The axis of the lateral femoral condyle of the femur was then determined by drawing a line between the most posterior and most anterior points of the lateral femoral condyle. The distance from the intersection of these two lines to the most inferior point of the lateral femoral condyle divided by the total anteroposterior length of the condyle to calculate the HAPR as follows: HAPR = H/AP (see Fig. 2). This ratio is used to describe the spherical shape of the lateral femoral condyle. We also measured the posterior tibial slope (PTS) [9, 10] to further evaluate the significance of the HAPR in predicting ACL injury (Fig. 2). We used two circles to determine the central axis of the proximal tibia. The proximal circle is 5 cm from the tibial plateau, and the distal circle is 5 cm from the proximal circle. The line passing through the center of the two circles was considered the long axis of the proximal tibia. The post tibial slope was defined as the angle formed by the axis that passes through the diaphyseal centre and a line parallel to the tibial plateau.

All measurements were performed by 2 blinded reviewers (Li RB, Fang Z) to determine interobserver reliability for the entire cohort. To assess intraobserver reliability, 1 reader (Li RB) repeated all measurements 1 month later.

### Statistical analysis

Quantitative data were expressed as the mean ± standard deviation. Statistical analyses were performed using SPSS software (version 24; IBM). A paired *t* test sample size estimation yielded a group size of 27 patients (alpha, 0.05; power, 0.8). Case-control matching was conducted according to gender (accurate matching) and age (maximum range of fluctuation up and down for 2 years). As the measurement results are nonnormal distribution, nonparametric test (Wilcoxon rank sum test) was used to analyze the differences between groups. Inter-and intraobserver reliabilities were measured by ICCs. The AUC was used to compare the two measurement methods, the optimal cutoff value was determined at the maximal Youden index [11].

## Results

**Fig. 3:**
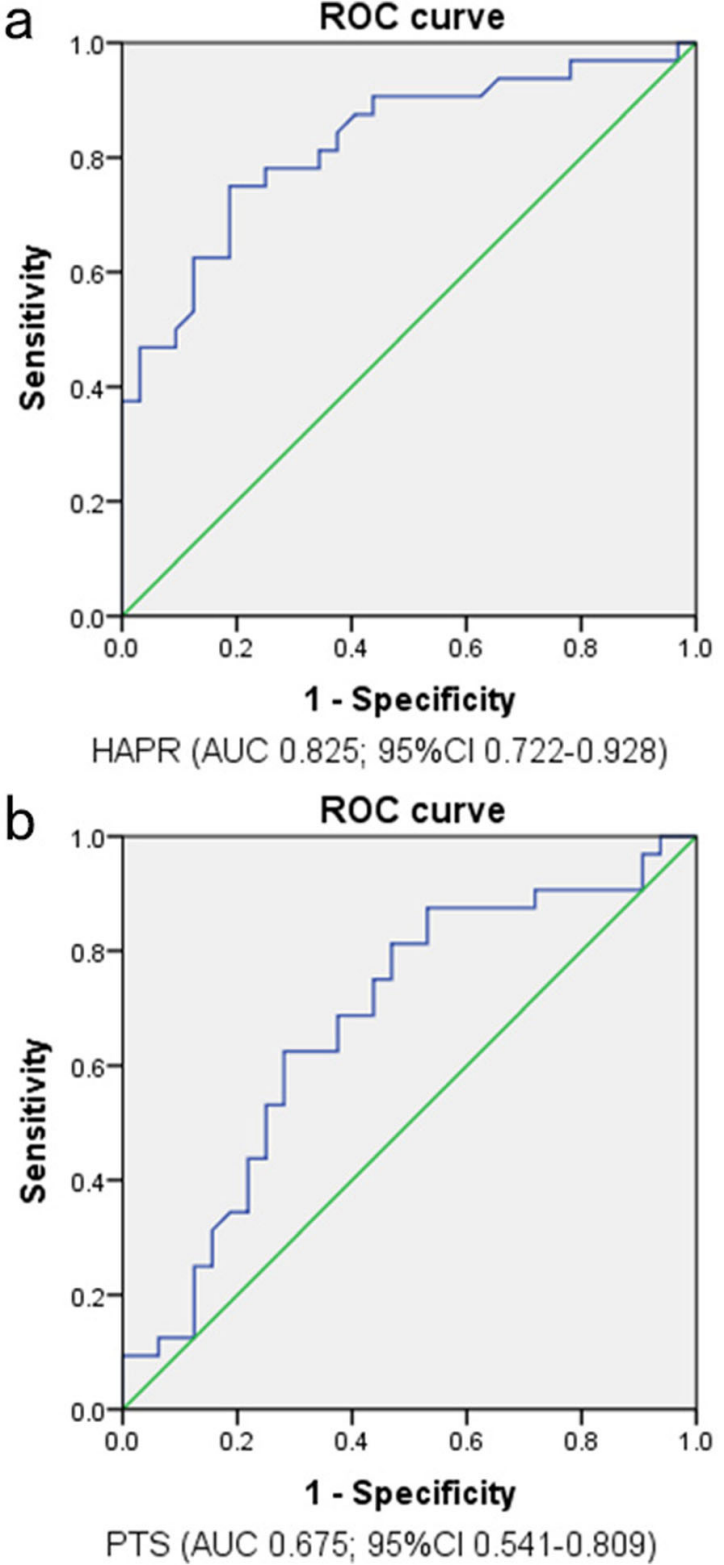
Receiver operating characteristic for (**a**) femoral and (**b**) tibial measurements. Reference line (diagonal): AUC = 0.5. AUC, area under the curve; HAPR, ratio of height of lateral femoral condyle to anteroposterior diameter; PTS, posterior tibial slope

Nine patients in the ACL rupture group were excluded due to malrotated radiographs, and twenty patients in the control group were excluded due to malrotated radiographs. A total of Sixty-four patients who met the inclusion criteria were included in the final analysis (32 with primary ACL rupture, 32 controls). Each group consisted of twenty-three male patients and nine female patients. The mean follow-up time was 36.2 ± 6.4 months in the ACL rupture group and 33.4 ± 5.5 months in the control group. The mean overlap of included radiographs was 3.25 ± 1.59 mm. The ICC for the HAPR was 0.86 (95% CI, 0.71–0.93) for the interobserver reliability and 0.95 (95% CI, 0.89–0.97) for the intraobserver reliability. The ICC for the PTS was 0.86 (95% CI, 0.70–0.93) for the interobserver reliability and 0.92 (95% CI, 0.84–0.96) for the intraobserver reliability (see Table 1). As shown in Table 2, there was no significant difference with regard to age (*p* = 0.813). The HAPR was smaller in the knees with primary ACL rupture (0.31 ± 0.02) than that of the control group (0.33 ± 0.02) (*p* < 0.01). The PTS was bigger in the knees with primary ACL rupture (8.18 ± 2.77) than that of the control group (6.61 ± 2.85) (*p* = 0.036). The AUC of HAPR was bigger (0.825; 95% CI, 0.72–0.93) than that of PTS (0.675; 95%CI, 0.85–0.81) (Fig. 3). The calculated cutoff of 0.32 (Youden index, 0.56) of HAPR was associated with an increased risk for ACL rupture, with sensitivity of 75% and specificity of 81% to predict an ACL rupture; (OR, 9.1; 95% CI, 2.9–28.5). The smaller the HAPR, the greater the risk of ACL rupture. The calculated cutoff of 7.1 (Youden index, 0.34) of PTS was associated with an increased risk for ACL rupture, with sensitivity of 63% and specificity of 72% to predict an ACL rupture; (OR, 4.3; 95% CI, 1.5–12.2). And the bigger the PTS, the greater the risk of ACL rupture.

**Table 1:** Inter- and intraobserver reliability among all DR measurements performed ^b^

	Intraobserver Reliability	Interobserver Reliability
HAPR	0.95(0.89–0.97)	0.86(0.71–0.93)
PTS	0.92(0.84–0.96)	0.86(0.70–0.93)

*HAPR* ratio of height of lateral femoral condyle to anteroposterior diameter, *PTS* posterior tibial slope

^b^Values are presented as intraclass correlation (95% CI). DR, digital radiograph imaging systems

**Table 2:** Demographics and measuring result of each study group

	ACL rupture	Control	*p*
N	32	32	
Age ^a^ (yr)	37 ± 10.8	37 ± 10.6	0.813
Male/female	23/9	23/9	
Follow-up ^a^ (mo)	36.2 ± 6.4	33.4 ± 5.5	
HAPR ^a^	0.31 ± 0.02	0.33 ± 0.02	< 0.01
PTS ^a^	8.18 ± 2.77	6.61 ± 2.85	0.036

HAPR, ratio of height of lateral femoral condyle to anteroposterior diameter. PTS, posterior tibial slope. *p* values refer to Wilcoxon rank sum test. *a* values are expressed as mean ± standard deviation

## Discussion

The most important finding of the present study is that a decreased HAPR is associated with an increased risk for an ACL rupture. HAPR helps to identify patients with a higher risk for ACL injuries, which proves the hypothesis of the study. A very robust cutoff value of 0.32 identified patients with an ACL rupture, with a sensitivity of 75% and a specificity of 81%.

Many studies have demonstrated the role of lateral femoral condyle morphology in the rotation mechanism of the knee joint [12, 13]. As the lateral femur rolls from its circular bending radius to its flatter part, the knee joint experiences physiologic rotational motion. Therefore, when the curvature radius of the lateral femoral condyle changes, the physiologic rotation function of the knee joint also changes. As Fernandes’s study [5] showed, a longer, flattened portion of the lateral femoral condyle of the femur is associated with ACL injury compared to the tibial plateau. This study represents the first to use the ratio of the height of the lateral femoral condyle to the anteroposterior diameters to evaluate the spherical shape of the lateral femoral condyle, and smaller HAPR means a smaller curvature femoral condyle. The study have shown that the smaller the HAPR, the higher the risk of ACL rupture. And this finding is consistent with Fernandes’s findings [5]. In addition, previous experiments [14] have confirmed that patients with ACL ruptured have a larger anteroposterior diameter of the lateral femoral condyle and a smaller height of the lateral femoral condyle, and consistent with the results of this study.

Patients with a decreased HAPR are at higher risk for an ACL rupture. This supports the hypothesis that patients with ACL injuries have increased rotation due to the influence of tibial and femoral bone morphology in the lateral compartment [15]. Increased pivoting has been shown to be associated with increased ACL strain and therefore contributes to an increased risk of ACL injury [16, 17].

Many studies, including this one, have confirmed that the steeper the tibial slope, the greater the risk of ACL injury, and some studies have calculated the threshold [14, 18, 19]. In this study, we found that the HAPR has better diagnostic and predictive value than the PTS.

This study has several advantages, but also some limitations. As advantages of this study, the presence of ACL rupture was confirmed by arthroscopy, which eliminated the possibility of diagnostic bias; it is presented a simple method that can be perform through lateral radiographs, which is low cost and less time consuming compared with other common radiography methods. On the other hand, limitations of this study include a reduction in the number of participants, especially female subjects; the need to exclude some radiographs due to poor quality.

## Conclusions

This study showed that a decreased HAPR is associated with an ACL rupture, and the decrease of HAPR was more significant in predicting ACL ruptures than the PTS. This helps clinicians identify susceptible individuals who may benefit from targeted ACL rupture prevention counseling and intervention.

## Data Availability

All raw data and materials during the study are available from the first author by request. (Li RB, lirb09@163.com).

## Abbreviations

ACL: Anterior cruciate ligament
HAPR: Ratio of height of lateral femoral condyle to anteroposterior diameter
PTS: Posterior tibial slope
DR: Digital radiograph imaging systems
ICC: Intracalss correlation coefficient
